# Medical image segmentation based on simulated annealing and opposition-based learning island algorithm

**DOI:** 10.1371/journal.pone.0307278

**Published:** 2024-07-24

**Authors:** M. A. JiMing, Duan HongYu, Wang YuFan, Wang LiNa

**Affiliations:** 1 School of Computer Science and Technology, Zhengzhou University of Light Industry, Zhengzhou, China; 2 School of Resource and Environmental Engineering, Jiangsu University of Technology, Changzhou, China; Islamia University of Bahawalpur: The Islamia University of Bahawalpur Pakistan, PAKISTAN

## Abstract

With the development of society and changes in the human living environment, people are increasingly attaching importance to their own health. Regarding medical imaging examinations of certain parts of the body, the process of medical image segmentation has become extremely important. This paper presents a novel hybrid algorithm: SAOBL-IA, a fusion of the Simulated Annealing(SA), Opposition-based Learning(OBL)and Island Algorithm(IA). The Island Algorithm itself suffers from slow convergence speed and the tendency to get stuck in local optimum. To address these limitations, we introduce opposition-based learning to enhance the search range and avoid local optimum. Furthermore, we leverage the simulated annealing approach to accelerate the convergence of SAOBL-IA. Comparing the experimental results, it can be seen that SAOBL-IA has better comprehensive performance. Subsequently, the SAOBL-IA algorithm is utilized in conjunction with an optimized two-dimensional OTSU fusion segmentation technique for the purpose of medical image processing. This study proposes an application of image segmentation based on the SAOBL-IA. The segmentation of pixels around the background and target regions using the two-dimensional OTSU method faces challenges in terms of accuracy. To address this issue, an adaptive thresholding technique known as Adaptive Forking is employed for optimization. By determining the slope of the fork based on the misclassified pixel ratio, enhanced segmentation accuracy can be achieved. This improved approach is then integrated with the SAOBL-IA algorithm and applied to the segmentation of lung medical images. The experimental findings show that the amalgamation of SAOBL-IA with the adaptive two-dimensional OTSU segmentation approach, as proposed in this study, manifests superior segmentation speed and enhanced precision in the context of medical image segmentation.

## 1. Introduction

Due to human activities, natural disasters, and other negative impacts on the atmospheric environment, such as air pollution caused by exhaust emissions, as well as personal bad habits such as long-term smoking and excessive drinking, etc. There has been a notable increase in the prevalence and mortality rates of pulmonary diseases [1]. Moreover, the emergence of highly contagious pulmonary diseases, such as novel coronaviruses, has further compounded the situation. Complicating matters, pulmonary diseases often exhibit subtle early symptoms, which can lead to their inadvertent neglect. Consequently, the timely detection and assessment of pulmonary diseases have become formidable challenges. Computed Tomography (CT), also known as electronic computed X-ray tomography, is currently the prevailing and efficacious modality for lung cancer detection and diagnosis [2]. Earlier iterations of CT scans yielded indistinct images that failed to delineate detailed pathological regions. Technological advancements have resulted in the production of significantly higher-resolution thinner CT slices, but have also increased the diagnostic burden. Integrating computer graphics technology for supportive diagnosis requires clear visualization of complex data. Through interactive modes, this technology aids physicians in detecting pathological areas, thus enhancing diagnostic accuracy.

A retrospective analysis of the developmental history of image segmentation techniques illustrates a progressive transition from manual segmentation to semi-automated segmentation and ultimately to fully automated segmentation. Image segmentation serves as a critical step in the realm of image analysis and has garnered significant utilization across numerous pertinent domains. Image segmentation can be broadly categorized into four major classes: region-based segmentation methods, comprising seed region growing and watershed algorithms; energy functional methods, including Snake models [3] and their derivatives; edge-based segmentation, including Roberts gradient operator [4], Sobel gradient operator, etc; and thresholding methods, such as the application of Two-dimensional(2D)OTSU in moon rocks and craters segmentation [5].

Image segmentation is essentially the foremost and elementary procedure to examine and construe the acquired image in innumerable computer vision applications wherein thresholding is considered enormously imperative in this domain [6], it can be mathematically modeled as an optimization problem, where swarm intelligence algorithms demonstrate excellent global search capability and rapid convergence speed, making them well-suited for addressing this problem. Consequently, an increasing number of scholars have started integrating these two approaches. The development of swarm intelligence algorithms has given rise to an increasing number of algorithms, such as the Island Algorithm (IA) [7], Particle Swarm Optimization (PSO) [8], Genetic Algorithm (GA) [9], Cuckoo Search (CS) [10], Firefly Algorithm (FA) [11], Grey Wolf Optimizer [12]. Researchers have applied various swarm intelligence optimization algorithms to the problem of image segmentation. For instance, in reference [13], a concise Binary Particle Swarm Optimization (BPSO) algorithm was proposed. This algorithm eliminates all parameters and directly obtains particle positions through Gaussian sampling. It combines with the OTSU method and is applied to parking space segmentation in the HSV color space. In reference [14], modifications were made to the original genetic algorithm by incorporating tournament selection procedure as the selection operator. Additionally, quantum bit mutation and quantum crossover operators were employed. In cases where the iterative performance was unsatisfactory, a catastrophe operation was initiated to ensure consistent convergence towards the optimal solution. The improved algorithm was then applied to multi-level image segmentation in the context of hyperspectral imagery [15, 16]. In order to tackle the challenges posed by weak inter-pixel correlations and ambiguous regions in satellite imagery, reference [17] integrates the Adaptive Cuckoo Search [18] algorithm with OTSU for image segmentation. The proposed algorithm enhances convergence towards optimal values by replacing Levy flight control step sizes with adaptive step sizes that are inversely proportional to the reproductive generation. This adaptive control mechanism effectively addresses the aforementioned issues.

This paper proposes a novel hybrid algorithm: SAOBL-IA, a fusion of the Simulated Annealing(SA) principles, Opposition-based Learning(OBL)ideas and Island Algorithm(IA). The SAOBL-IA approach is designed to increase the algorithm’s ability to escape local optima and accelerate convergence speed. Furthermore, it is combined with an improved 2D OTSU algorithm to overcome the slow segmentation speed and misclassification of background and peripheral target pixels. The proposed method is applied to medical image processing of lung images, and simulation results indicate that the combination of SAOBL-IA and adaptive 2D OTSU has faster image segmentation speed and higher accuracy.

## 2. Island algorithm

The Island Algorithm (IA) is a novel metaheuristic algorithm that mimics the adaptive survival of plants on islands in response to changes in sea level in nature. The algorithm continuously raises the sea level, while the total number of surviving plants remains constant, in order to search for the most suitable location for survival. The Island Algorithm consists of three stages: Elimination stage, Sea level rising stage, and Equilibrium stage.

### 2.1 Elimination stage

The primary objective of the elimination stage is to determine the number of individuals to be eliminated in the current iteration based on the variation in the island range, denoted as h. To defining a range necessitates the establishment of upper and lower bounds, requiring a minimum of two plants to delineate the extent of the island. Consequently, the maximum elimination count should be less than the total number of plants minus 2. Simultaneously, when the variation in the island range is significant, the algorithm is prone to getting trapped in local optimum. Therefore, it is necessary to reduce the number of eliminations in the next iteration to decrease the range variation in subsequent iterations. Conversely, when the variation in the island range is small, the algorithm exhibits slower convergence. In such cases, increasing the number of eliminations can be employed to amplify the range variation in the next iteration. Hence, the elimination function adheres to the following two criteria:

1. When the range variation is minimal (i.e., when the range variation is 0), the maximum number of individuals to be eliminated is required (i.e., the total number of plants minus 2).
2. The overall elimination function is a decreasing function.

Therefore, a negative exponential function that meets the above two criteria and is relatively straightforward can be used:

$$f_{weedout}(h)=[(A_{max}-A_{min})\cdot e^{-h}]+A_{min}$$ (1)

In Eq (1): h represents the range variation with an initial value of 1, *A*_*max*_ denotes the maximum number of eliminations, and *A*_*min*_ corresponds to the minimum number of eliminations.

### 2.2 Sea level rise stage

During the phase of rising sea levels, plant elimination is carried out based on the number of eliminations generated in the preceding stage. This elimination process is characterized by a concomitant reduction in the island range due to the ascending sea levels. The primary objective of this stage is to generate novel island ranges and corresponding range variations. The new island range serves as input for the subsequent equilibrium phase, whereas the newly obtained range variation is utilized in the elimination phase of the ensuing iteration.

The determination of the new island range is based on the maximum and minimum values of non-eliminated plants, where X_max_ and X_min_ denote the maximum and minimum values for each dimension, respectively. To avoid premature convergence of the algorithm, it is essential to extend the maximum and minimum values of the range using Formulas (2) and (3):

$$X_{max}=X_{max}+rand\cdot (X_{max}^{old}-X_{max})$$ (2)

$$X_{min}=X_{min}+rand\cdot (X_{min}^{old}-X_{min})$$ (3)

The formula for the variation in island range is given as Formula (4):

$$h=norm\left((X_{max}^{old}-X_{min}^{old})-(X_{max}-X_{min})\right)$$ (4)

Among them: X_max_ represents the maximum value for each dimension in the current iteration, while X_min_ represents the minimum value for each dimension. On the other hand, $X_{max}^{old}$ and $X_{min}^{old}$ represent the maximum and minimum values for each dimension in previous iteration. The function "norm" is used to calculate the 2-norm between vectors.

### 2.3 Equilibrium stage

During the equilibrium phase, the main task is to shrink the island range while preserving the total number of plants. This is achieved by eliminating old plants and simultaneously introducing an equal number of new plants. The non-eliminated old plants remain unchanged in their original positions, while the newly generated plants strive towards optimal global positions. At the conclusion of the equilibrium phase, fitness values are compared and sorted. The formula for generating new plants is expressed as Formula (5).


x(j,:)=x(j,:)+2×rand(1,D)·(x(1,:)−x(j,:)) (5)

In Eq (5): x(j,:) represents the position of the j-th new plant within the new range; x(1,:) denotes the globally optimal position; and D signifies the dimensionality.

## 3. Improved island algorithm

Although the original island algorithm boasts advantages such as fast convergence speed and robustness, it encounters certain issues during the equilibrium phase. Specifically, the algorithm’s utilization of a fixed plant position iteration method leads to incomplete exploration of the current search space. Furthermore, when confronted with overly flat regions or sharp peak functions, the original island algorithm may struggle to explore optimal solutions effectively. Hence, this paper introduces an opposition-based learning approach to iteratively update excellent plants and combines it with a simulated annealing algorithm to comprehensively explore local space and enhance the exploration of global optimal solutions.

### 3.1 Opposition-based learning

**Fig 1:**
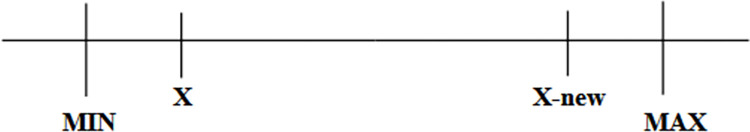
Opposition-based learning.

The thought of Opposition-based Learning [19] is to evaluate the opposition-based learning solution based on the current feasible solution, and select the better feasible solution [20]. Fig 1 provides an illustration of this process.

In the Fig 1, MAX and MIN represent the range of values for the independent variable. X-new corresponds to the opposition-based learning point of X, and its value is calculated as MAX + MIN—X. Therefore, when generating new positions in the algorithm, the opposition-based learning position is simultaneously selected and compared with the fitness value of the original position, to choose the optimal solution. In reference [21], the opposition-based learning technique is integrated into the Particle Swarm Optimization algorithm. When the optimal value of the iteration remains unchanged for an extended period, a reverse search is conducted by selecting suboptimal particles and maximizing their flight velocity, thereby effectively avoiding premature convergence. Additionally, in reference [22], the concept of antagonistic learning is incorporated into the Hybrid Jumping Frog Algorithm, whereby reverse learning is applied to the elite group. This entails recalculating the positions of the best-performing frog group in the entire solution space in a reverse manner. Such an approach expands the search algorithm’s neighborhood search capability, enabling it to circumvent local optima and achieve enhanced precision. The iCSPM2 [23] algorithm improves the cuckoo search algorithm by initially dividing particles into four islands using an island strategy [24], followed by employing different evolutionary strategies for each of the four islands, resulting in four improved versions of the cuckoo search algorithm. Finally, the elite reverse learning strategy is incorporated at the end of each round to retain better particles. Similarly, DiCSPM [25] also enhances the cuckoo search algorithm, specifically its parallel version iCSPM, using the reverse learning method, albeit applied to the island strategy where each island contains the opposite population of another island, unlike the aforementioned literature. In contrast, IBSCA3 [26] primarily enhances the Binary Sine Cosine Algorithm (SCA) starting from the IBSCA1 version. While IBSCA1 improves through reverse learning, IBSCA2 additionally utilizes the VNS method and the Laplace distribution. IBSCA3 further incorporates refraction learning during iteration to enhance exploration capabilities. Although these papers employ reverse learning algorithms, the underlying algorithms and application areas differ, differing from the focus of our study.

### 3.2 Simulated annealing

The Simulated Annealing method was originally used to solve combinatorial optimization problems [27]. It is primarily based on simulating the annealing process of objects, where the object is heated to increase molecular activity and induce disorder, and then gradually cooled down to reach an equilibrium state. The algorithm primarily utilizes the temperature parameter to regulate its progression towards local or global optima. At the outset, when the temperature is sufficiently high, the algorithm can tolerate suboptimal deteriorations. As the annealing process unfolds, the temperature gradually decreases, causing the algorithm to selectively favor superior solutions. Eventually, at a temperature of 0, only the most optimal solutions are accepted, leading to the eventual attainment of either a local or global optimum. The selection method employed in the Simulated Annealing Algorithm is elucidated as follows:

$$p=\left\{ \begin{array}{l} \;1,\;E_{2}<E_{1}\\ \exp\left(-\frac{E_{2}-E_{1}}{T}\right),\;E_{2}\geq E_{1} \end{array}\right.$$ (6)

In Eq (6), p represents the probability of accepting a new solution, while E denotes the fitness value associated with the solution. When *E2 < E1*, the new solution is accepted due to its superior fitness value. However, when *E*_*2*_ is greater than or equal to *E*_*1*_, there exists a certain probability of accepting deteriorating solutions, thus facilitating more effective global exploration. Furthermore, literature has integrated simulated annealing with Gaussian mutation in the Particle Swarm Optimization algorithm to select mutated particles, thereby enhancing convergence speed and precision. Reference [28] leverages the early non-convergence advantage of simulated annealing and integrates it with genetic algorithms to overcome the issue of premature convergence, thereby enhancing the overall capability of the algorithm.

### 3.3 The SA and OBL island algorithm (SAOBL-IA)

During the iteration phase of the equilibrium stage in the Island Algorithm, not all plant positions are updated. Instead, only inferior plants are eliminated while retaining the existing superior plants. Although this approach reduces the computational burden to some extent, it results in incomplete exploration of the search space and slower convergence speed, thus impacting the algorithm’s search capability. Hence, the present study incorporates the idea of opposition-based learning to tackle the aforementioned challenges by updating the positions of the existing retained plants through opposition-based learning. The selection of preserved plants is based on a comparison between the fitness values of original plant positions and their corresponding counterparts derived from opposition-based learning. This method not only retains superior plants but also introduces novel ones, thereby speeding up the overall convergence rate of the algorithm. The formula for generating new positions is expressed as Eq (7).


$$x_{new}=\left\{ \begin{array}{l} \;x_{old},\;f(a+b-x_{old})>f(x_{old})\\ \left(a+b-x_{old}),\;f(a+b-x_{old})\leq f(x_{old}\right) \end{array}\right.$$ (7)

In Eqion (7), *x*_*new*_ corresponds to the selected position after opposition-based learning, *x*_*old*_ represents the original solution, and (*a*+*b*−*x*_*old*_) denotes the opposition-based learning positions within the island range. For instance, when the island range is between 0 and 10, with a being 0 and b being 10, if the old position of a plant is *x*_*old*_ = 3, then its opposition-based learning position would be (*a*+*b*−*x*_*old*_) = 7. Here, *a* and *b* represent the upper and lower bounds on the island, respectively. The function f denotes the fitness function. If the fitness value of the new position is superior to that of the old position, a replacement is performed.

**Fig 2:**
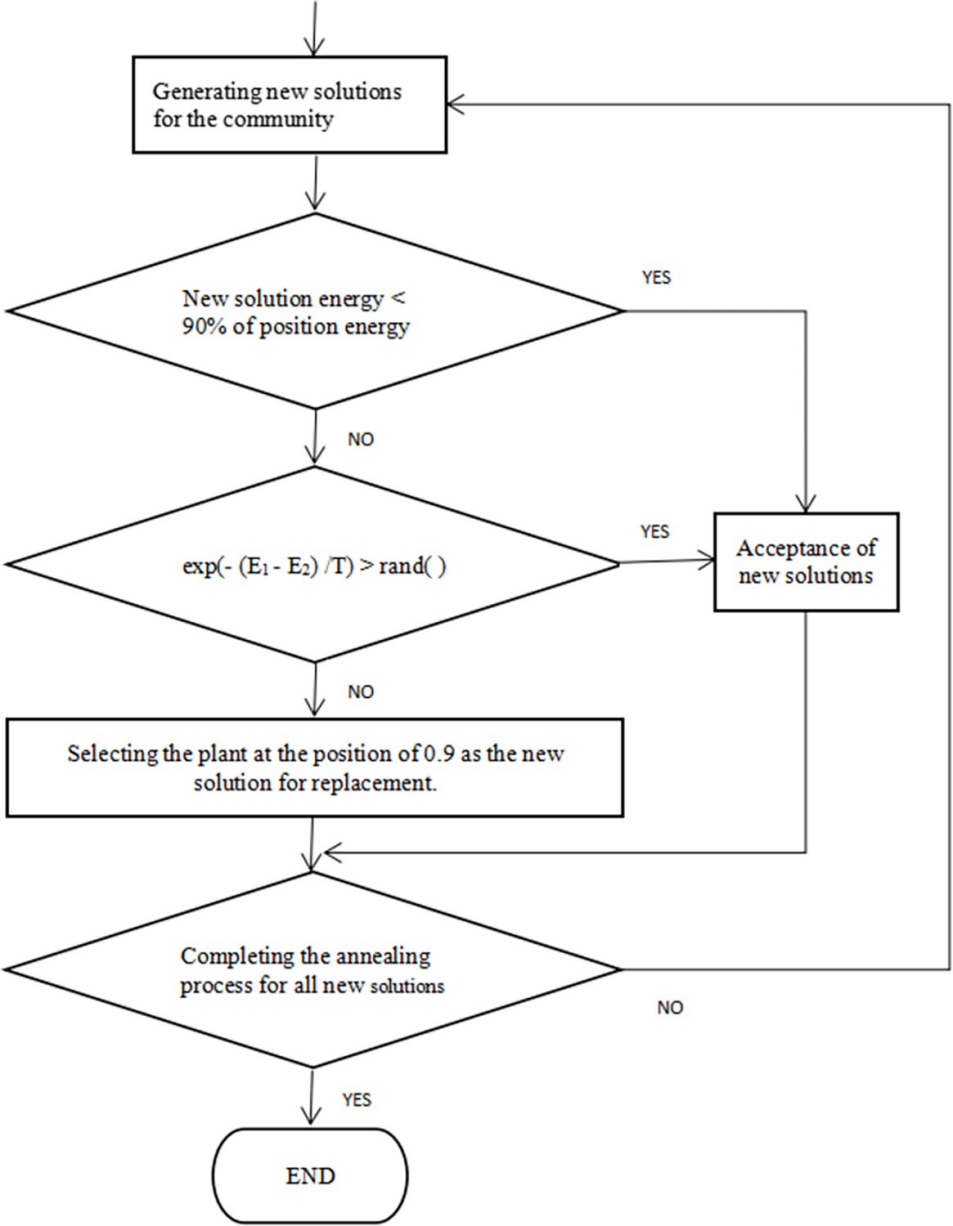
The generation process of new solutions during the equilibrium phase.

In addition, the Island Algorithm often encounters slow convergence or difficulty in exploring the optimal solution when dealing with excessively flat regions or sharp peaks. The present study incorporates the concept of simulated annealing in the selection of newly generated plants. When the Island Algorithm generates new island ranges during the ascent phase, the 90th percentile position of the non-eliminated plants is selected as the threshold for the annealing process. If the annealing energy is lower than the energy at this position, the new position is adopted; otherwise, a certain probability-based judgment is made according to temperature conditions. When the conditions are not met, the new position is replaced with the plant position at the threshold (as shown in Fig 2). At the beginning of the iteration, energy levels are accepted regardless of their values; however, as the iteration progresses and the temperature decreases, only better solutions are accepted. Therefore, this approach can more comprehensively search the solution space and accelerate the convergence speed.

### 3.4 Algorithm steps

The steps of the Island Algorithm based on SA and OBL are as follows.

Step 1 Initialize the island algorithm with a random set of solutions, population size N = 100, dimension D, maximum number of iterations T = 1500, maximum number of evaluations E, The maximum and minimum number of eliminations A_max_ = 50, A_min_ = 2, island range h = 1, simulated annealing initial temperature t = 100(The maximum elimination count is typically determined based on the population size, while the selection of the initial temperature and the cooling coefficient are derived from multiple experimental tests.).

Step 2 Compute individual fitness values and sort them, while selecting the best fitness value and its corresponding position.

Step 3 During the iteration process, the elimination procedure is executed according to Formula 1, generating the required number of individuals to be eliminated.

Step 4 In the sea level rise phase, new island ranges are generated and expanded according to Formulas 2 and 3, and the variation of island range is calculated based on Formula 4.

Step 5 In the balancing phase, the retained plants are subjected to opposition-based learning as per Formula 7. The resulting fitness values are then compared, and the more favorable plants are selected for replacement.

Step 6 The novel vegetation obtained from Formula 5 is subjected to the simulated annealing algorithm, utilizing Formula 6 for selection purposes. The enhanced vegetation generated from the integration of the two methods is then used to create the succeeding generation, with annealing being carried out subsequently.

Step 7 Check if the algorithm meets the termination criteria. Reaching the maximum number of iterations or attaining the theoretical optimum value of the optimization function during the iterative process. If the termination criteria are met, output the results and end the algorithm. Otherwise, continue looping by executing step 3. The pseudo-code for the algorithm is shown below:

**SAOBL-IA pseudo-code**

Input: Initialization N, A_max_, A_min_, h, D, T, E, t.

 Island location: $X_{max}=100\cdot ones(1,D),X_{min}=-100\cdot ones(1,D)$

Plant location: $x=ones(N,1)\cdot X_{min}+ones(N,1)\cdot (X_{max}-X_{min})\cdot rand(N,D)$

 Calculating and ranking the fitness values for each plant,

   [fitness,index]=sort(fit),x=x(index,:)

Output: Optimum value location:*x*(1,:*x*), Optimal fitness value:*fit*(1);

  while (termination condition)

elimination phase:

  Calculate the number of eliminated individuals using Formula 1

sea level rise phase:

$X_{max}=max(x(1:N-tt,:));X_{min}=min(x(1:N-tt,:))$

   Expand the range according to Formulas 2 and 3.

Using Formula 4 to calculate the change in island range h.

   Balancing phase:

Opposition-based Learning is performed on the old plants,

  the reverse position x_opp_ is calculated according to Formula 7,

   and the fitness value fit_opp_ is calculated.

  for (All the old plants)

   if (fit_opp_<fit_old_)

Replace with the corresponding reverse position and fitness value.

   end

  end

Generating new plant locations according to Formula 5.

Perform simulated annealing on the new plant and

select mid = round((N−out)*0.9) as the annealing critical point.

  for (All the new plants)

   if (E>0)

 if (Decide whether to accept worse solutions according to Formula 6)

Replace the poorer solution with mid.

    end

   end

  end

 t = t*0.99;

 if (reaching the maximum evaluation times)

Produce the output and terminate the algorithm.

 else

increment the evaluation count.

 end

[fit,index]=sort(fit),x=x(index,:);

 end

## 4. Lung image segmentation based on SAOBL-IA

In response to the slow processing speed of traditional two-dimensional Otsu segmentation and the potential misclassification of pixels along the diagonal during segmentation, this study introduces an enhanced version of the island algorithm. The enhanced algorithm aims to improve both the speed and accuracy of finding the optimal threshold value, and enhances the two-dimensional Otsu method by employing a bifurcation approach, thereby strengthening its ability to segment pixels along the diagonal.

### 4.1 Traditional 2D OTSU algorithm

The traditional 2D maximum inter-class variance algorithm utilizes pixel grayscale and its neighborhood grayscale mean to construct a 2D histogram for threshold segmentation. Due to the lack of utilization of pixel local spatial neighborhood information, one-dimensional(1D)OTSU cannot achieve ideal results or even result in incorrect segmentation when the grayscale difference between the background and the target is not significant [29]. The added 1D data compensates for the correlation information between pixels, which is beneficial for restraining noise and improving segmentation accuracy. However, the additional incorporation of local grayscale value information necessitates the calculation of the mean for each pixel’s surroundings, thereby augmenting the algorithm’s time complexity. This poses significant challenges to computational resources and utilization efficiency. As pointed out in reference [30], the current application of swarm intelligence algorithms in image segmentation still faces numerous challenges. For instance, the efficiency of the objective function largely depends on both the swarm intelligence algorithm employed and the type of image being segmented. By combining the global search of particle swarm optimization algorithm with the local search of bat algorithm, the 2D OTSU threshold is optimized, which improves the computational speed and storage space [31]. By focusing on the average of pixel grayscale values and neighborhood grayscale values in the central region of the image, the optimal threshold is found, and the binary quantum particle swarm optimization algorithm is integrated to improve the quality of segmented images [32]. A threshold point is determined by intersecting the diagonal and perpendicular lines on the 2D histogram, which considers the pixels along the diagonal but also introduces redundant pixels [33]. In reference [34], The main approach employs the ES-WOA algorithm, which itself utilizes the strategy of Evolution Strategies (ES). This strategy involves conducting a global search using Levy flights initially, forming a new population of better solutions. Subsequently, techniques such as Firefly Algorithm (FA), Differential Evolution (DE), and Particle Swarm Optimization (PSO), among others, are employed as local optimizers to update and iterate over the new population. Conversely, this paper adopts the Cauchy distribution for global search and the Whale Optimization Algorithm (WOA) as the local search mechanism. Moreover, the improved algorithm is integrated into various image segmentation algorithms, including the application of Otsu in multi-level color hematology image segmentation. In reference [35, 36], claimed to achieve better optimization capabilities by employing the Hybrid Equilibrium Optimization Algorithm (HEOA) in 3D Otsu segmentation of standard color images and color satellite images from the CEC2015 dataset for multi-level image segmentation. However, the 3D segmentation algorithm also requires significant computational resources, resulting in substantial time consumption. In reference [37], an improved adaptive differential evolution algorithm is combined with the 2D maximum inter-class variance algorithm to efficiently obtain the threshold value.

The principle of the 2D OTSU algorithm is: Let M*M be the size of the image with L levels of gray scale for its pixels, and the same L levels for its neighborhood gray scale average. The total number of pixels is N, where f(x,y) represents a pixel with gray scale i and neighborhood gray scale average j, and they form a tuple. The number of occurrences of a certain tuple is denoted by f_ij_ and its probability by P_ij_.then:

$$P_{ij}=f_{ij}/N$$ (8)

$$\sum_{i=0}^{L-1}\sum_{j=0}^{L-1}P_{ij}=1,\sum_{i=0}^{L-1}\sum_{j=0}^{L-1}f_{ij}=N$$ (9)

**Fig 3:**
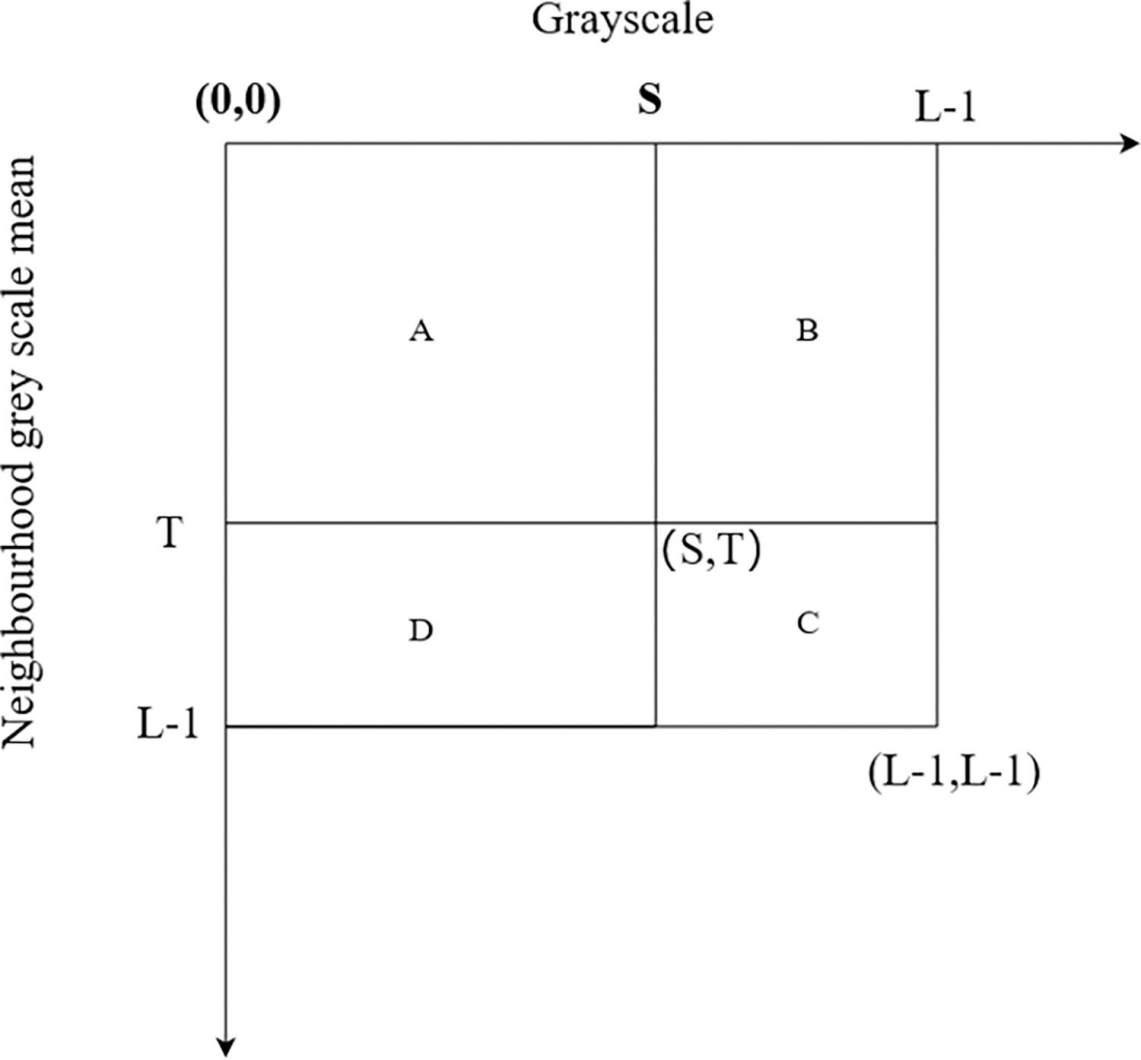
Two-dimensional histogram plane.

Choosing a threshold vector *(s*, *t)* divides the two-dimensional histogram of Fig 3 into four regions, A, B, C, and D. A and C respectively represent the background and object parts of the image, while B and D represent the edge and noise. The probabilities of occurrence of the background and object are then given by:

$$\omega_{1}=\sum_{i=0}^{s}\sum_{j=0}^{t}P_{ij}\;,\omega_{2}=\sum_{i=s+1}^{L-1}\sum_{j=t+1}^{L-1}P_{ij}$$ (10)

The mean vectors for the background and target are:

$$\mu_{1}={\left(\mu_{1i},\mu_{1j}\right)}^{T}={\left(\sum_{i=0}^{s}\sum_{j=0}^{t}\frac{iP_{ij}}{\omega_{1}},\sum_{i=0}^{s}\sum_{j=0}^{t}\frac{jP_{ij}}{\omega_{1}}\right)}^{T}$$ (11)

$$\mu_{2}={\left(\mu_{2i},\mu_{2j}\right)}^{T}={\left(\sum_{i=s+1}^{L-1}\sum_{j=t+1}^{L-1}\frac{iP_{ij}}{\omega_{2}},\sum_{i=s+1}^{L-1}\sum_{j=t+1}^{L-1}\frac{jP_{ij}}{\omega_{2}}\right)}^{T}$$ (12)

The total mean vector of the two-dimensional histogram is:

$$\mu_{T}={\left(\mu_{Ti},\mu_{Tj}\right)}^{T}={\left(\sum_{i=0}^{L-1}\sum_{j=0}^{L-1}iP_{ij},\sum_{i=0}^{L-1}\sum_{j=0}^{L-1}jP_{ij}\right)}^{T}$$ (13)

In the 2D maximum inter-class variance algorithm, it is common to ignore the probabilities of regions B and D due to their relatively low values. Therefore, they can be set as negligible: *ω*_1_+*ω*_2_ = 1 and $\mu_{T}=\omega_{1}\mu_{1}+{\omega_{2}\mu }_{2}$.

Inter-class variance is defined as:

$$\sigma =\omega_{1}[{\left(\mu_{1i}-\mu_{Ti}\right)}^{2}+{\left(\mu_{1j}-\mu_{Tj}\right)}^{2}]+\omega_{2}[{\left(\mu_{2i}-\mu_{Ti}\right)}^{2}+{\left(\mu_{2j}-\mu_{Tj}\right)}^{2}]$$ (14)

Therefore, the optimal threshold is the value of (s, t) when σ reaches its maximum value.

### 4.2 A modified adaptive bifurcation 2D OTSU method

For the traditional 2D maximum inter-class variance algorithm, when the threshold selection is near the main diagonal, the probability in the main diagonal region can be approximated to 1. However, when the threshold is far from the main diagonal and near the secondary diagonal, the approximation of the probability to 1 no longer holds, as the probability is affected by noise and edge interference. If the segmentation is still performed according to the traditional method, segmentation errors will occur, affecting accuracy and preventing the attainment of the optimal threshold. In order to solve this problem, we propose a modified Adaptive Bifurcation 2D OTSU(AB2D-OTSU) technique as follows.

In actual experiments, we found that there are still many non-zero tuples in the binary tuples close to the background and object regions, which may be due to noise interference resulting in deviation and should be included in the background or object regions. Therefore, the bivariate histogram plane is divided by two lines, *l*_1_ and *l*_2_, passing through the threshold point *(s*, *t)*, where the angle between line *l*_1_ and the line *x = s*, and the angle between line *l*_2_ and the line *y = t*, are both α and (0°≤α≤5°). The expression is:

$$l_{1}:y=(s-x)cot\alpha +t$$ (15)

$$l_{2}:y=(s+x)cot\alpha +t$$ (16)

**Fig 4:**
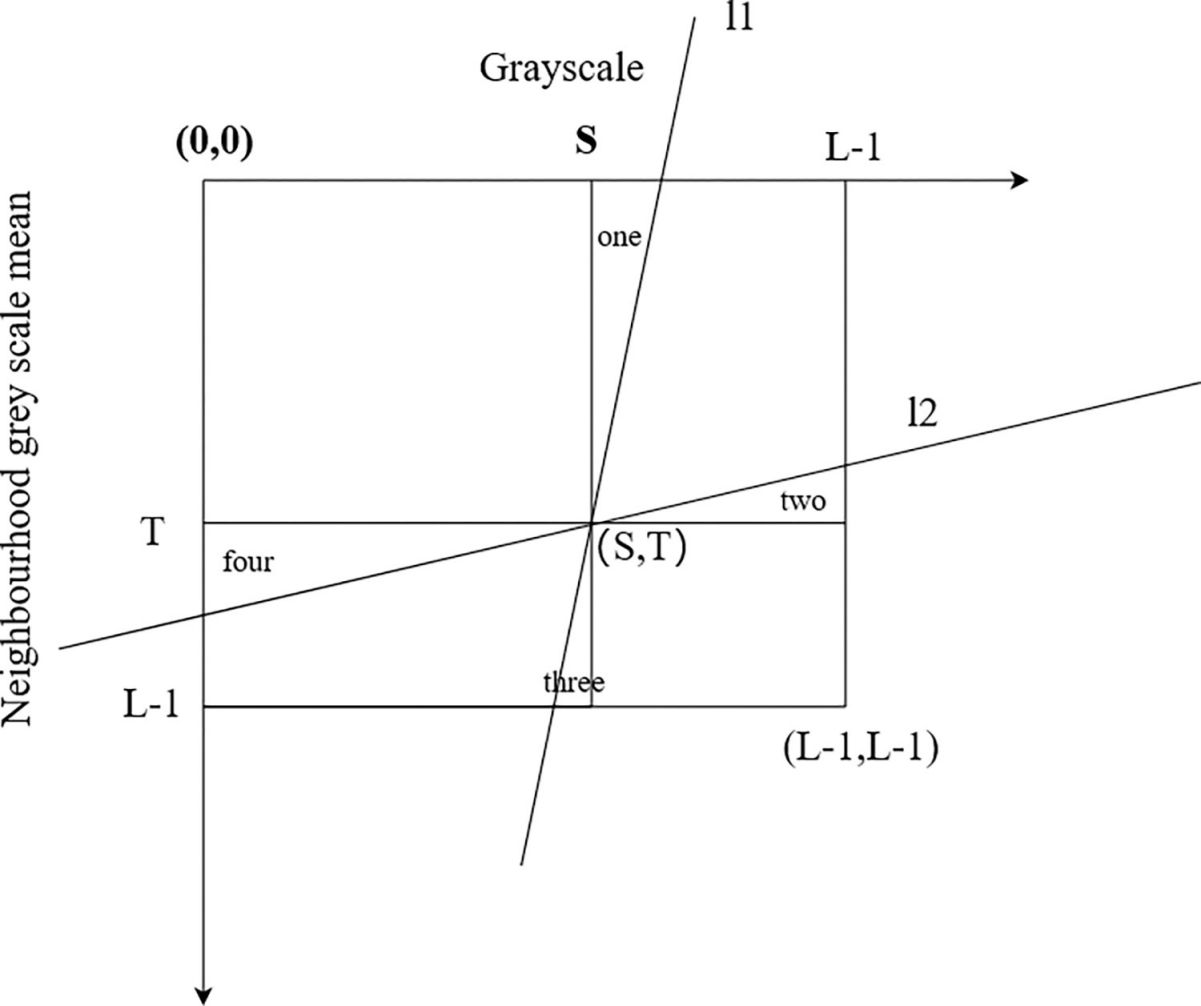
Adaptive bifurcation histogram plane.

According to Fig 4, the four regions (designated as one, two, three, and four) are enclosed by the two lines and the background and object regions. The zero-element tuples within the enclosed area are denoted as N_0_, while the non-zero-element tuples are denoted as N_α_, and N_α_ proportion is denoted as P_α_. When P_α_ = 0.75, α is fixed. Through this approach, pixels along the diagonal, which are susceptible to misclassification as either background or foreground, are systematically classified, thereby enhancing the accuracy of image segmentation. If there are no suitable angles in 0°≤α≤5°, the enclosed region is abandoned as the background and object and the calculation is performed on the original region. among:

$$P_{\alpha }=\frac{N_{\alpha }}{N_{\alpha }+N_{0}}$$ (17)

The updated probabilities for the foreground and background are as follows:

$$\omega_{\alpha 1}=\sum_{i=0}^{s}\sum_{j=0}^{t}P_{ij}+\sum_{i=s}^{s+t\tan\;\alpha }\sum_{j=0}^{l_{1}}P_{ij}+\sum_{i=0}^{t}\sum_{j=t}^{l_{2}}P_{ij}$$ (18)

$$\omega_{2}=\sum_{i=s+1}^{L-1}\sum_{j=t+1}^{L-1}P_{ij}+\sum_{i=s}^{s+t\tan\;\alpha }\sum_{j=0}^{l_{1}}P_{ij}+\sum_{i=0}^{t}\sum_{j=t}^{l_{2}}P_{ij}$$ (19)

The updated mean vectors for the two categories are as follows:

$$\mu_{\alpha 1}={\left(\mu_{\alpha 1i},\mu_{\alpha 1j}\right)}^{T}={\begin{array}{c} (\sum_{i=0}^{s}\sum_{j=0}^{t}\frac{iP_{ij}}{\omega_{\alpha 1}}+\sum_{i=s}^{s+t\tan\;\alpha }\sum_{j=0}^{l_{1}}\frac{iP_{ij}}{\omega_{\alpha 1}}+\sum_{i=0}^{t}\sum_{j=t}^{l_{2}}\frac{iP_{ij}}{\omega_{\alpha 1}},\\ \sum_{i=0}^{s}\sum_{j=0}^{t}\frac{jP_{ij}}{\omega_{\alpha 1}}+\sum_{i=s}^{s+t\tan\;\alpha }\sum_{j=0}^{l_{1}}\frac{jP_{ij}}{\omega_{\alpha 1}}+\sum_{i=0}^{t}\sum_{j=t}^{l_{2}}\frac{jP_{ij}}{\omega_{\alpha 1}}) \end{array}}^{T}$$ (20)

$$\mu_{\alpha 2}={\left(\mu_{\alpha 2i},\mu_{\alpha 2j}\right)}^{T}={\begin{array}{c} (\sum_{i=s+1}^{L-1}\sum_{j=t+1}^{L-1}\frac{iP_{ij}}{\omega_{\alpha 2}}+\sum_{i=s+1-t\tan\;\alpha }^{s+1}\sum_{j=t+1}^{l_{1}}\frac{iP_{ij}}{\omega_{\alpha 2}}+\sum_{i=s+1}^{L-1}\sum_{j=l_{2}}^{t+1}\frac{iP_{ij}}{\omega_{\alpha 2}},\\ \sum_{i=s+1}^{L-1}\sum_{j=t+1}^{L-1}\frac{jP_{ij}}{\omega_{\alpha 2}}+\sum_{i=s+1-t\tan\;\alpha }^{s+1}\sum_{j=t+1}^{l_{1}}\frac{jP_{ij}}{\omega_{\alpha 2}}+\sum_{i=s+1}^{L-1}\sum_{j=l_{2}}^{t+1}\frac{jP_{ij}}{\omega_{\alpha 2}}) \end{array}}^{T}$$ (21)

The updated equation for inter-class variance in two dimensions is as follows:

$$\sigma_{\alpha }=\omega_{\alpha 1}[{\left(\mu_{\alpha 1i}-\mu_{Ti}\right)}^{2}+{\left(\mu_{\alpha 1j}-\mu_{Tj}\right)}^{2}]+\omega_{\alpha 2}[{\left(\mu_{\alpha 2i}-\mu_{Ti}\right)}^{2}+{\left(\mu_{\alpha 2j}-\mu_{Tj}\right)}^{2}]$$ (22)

### 4.3 Image segmentation algorithm based on SAOBL-IA

To address the issue of high computational complexity and long processing time of the improved two-dimensional maximum inter-class variance algorithm, we propose to use the SAOBL-IA to enhance image segmentation algorithm speed and obtain the optimal threshold for segmentation. The main steps of the algorithm are as follows:

Setp1 Read the lung image to be segmented, initialize the algorithm parameters: population size N, dimensionality D, maximum number of iterations T, maximum number of evaluations E, maximum number of eliminations A_max_, minimum number of eliminations A_min_, and initial temperature *t* of the simulated annealing.

Setp2 Calculate the initial maximum two-dimensional inter-class variance corresponding to the threshold, and sort to select the maximum variance value.

Setp3 Perform opposition-based learning on the segmentation threshold, select a better solution, and eliminate some of the poorer solutions.

Setp4 Supplement the eliminated solutions through simulated annealing.

Setp5 Iterate to optimize according to Steps 3 and 4, determine whether the algorithm meets the termination conditions, output the optimal solution, that is, the optimal threshold binary tuple, if yes, otherwise continue iterating.

Setp6 Segment the image based on the optimal threshold binary tuple and output the image.

## 5. Experiments and analysis of experimental results

This chapter aims to test the overall performance of the improved island algorithm. By employing seven benchmark test functions, we will evaluate the algorithm’s robustness, ability to escape local optima, and global search capabilities. Additionally, we will examine the effectiveness of integrating the improved algorithm with the bifurcation-based two-dimensional Otsu algorithm, and validate the superiority of the improved algorithm through metrics such as segmentation accuracy.

### 5.1 Experimentation and analysis of the SAOBL-IA

#### 5.1.1 Experimental design for the performance of the SAOBL-IA

In order to verify the overall performance improvement of the Island Algorithm, we selected seven typical benchmark functions to test the algorithm’s performance. The specific information of the functions is shown in Table 1. Here *f*_*1*_ is a spherical multidimensional single-peak function with multiple local minima besides the global minimum, and its main purpose is to test the algorithm’s optimization ability. *f*_*2*_ is a multi-modal function with a valley-like shape and multiple local minima, and *f*_*6*_ has multiple global minima, mainly designed to test the global search ability of the algorithm. *f*_*3*_ is a highly multimodal function and, similar to *f*_*4*_, has many regularly distributed local minima, which are used to test the algorithm’s ability to escape from local optima. The main purpose of testing function *f*_*5*_ and unimodal function *f*_*7*_ is to evaluate the algorithm’s convergence ability. The experiment compared Particle Swarm Optimization algorithm (PSO), Beetle Antennae Search algorithm (BAS), Island Algorithm (IA), Grey Wolf Optimizer(GWO) and Island Algorithm based on Simulated Annealing and opposition-based learning (SAOBL-IA). The five algorithms were independently run 30 times on 30-dimensional and 50-dimensional problems for comparison. The best, worst, average, and standard deviation of the optimal error values obtained from 30 independent runs of PSO, BAS, IA, GWO and SAOBL-IA on 30 and 50 dimensions were used as the evaluation criteria for algorithm performance, and the bold font in the table represents the best performance. All five algorithm parameters are set as follows: population size 100, The algorithms are all in Matlab R2016a software for Windows 10 with an i7-11800H 2.3GHz CPU and 16GB of RAM.

**Table 1:** Specific information on the 7 benchmark functions.

function name	Formula	Scope
***f*_1_.Sphere**	$f_{1}(x)=\sum_{i=1}^{d}x_{i}^{2}$	[−5.12,5.12]^D^
***f*_2_.Dixon**	$f_{2}(x)={\left(x_{1}-1\right)}^{2}+\sum_{i=2}^{d}i{\left(2x_{i}^{2}-x_{i-1}\right)}^{2}$	[−10,10]^D^
***f*_3_.Rastrigin**	$f_{3}(x)=10d+\sum_{i=1}^{d}\left[x_{i}^{2}-10cos(2\pi x_{i})\right]$	[−5.21,5.12]^D^
***f*_4_.Griewank**	$f_{4}(x)=\sum_{i=1}^{d}\frac{x_{i}^{2}}{4000}-\prod_{i=1}^{d}\cos\left(\frac{x_{i}}{\sqrt{i}}\right)+1$	[−600,600]^*D*^
***f*_5_.Salomon**	$f_{5}(x)=-\cos\left(2\pi \sqrt{\sum_{i=1}^{d}x_{i}^{2}}\right)+0.1\sqrt{\sum_{i=1}^{d}x_{i}^{2}}+1$	[−10,10]^*D*^
***f*_6_.Branin**	$f_{6}(x)={\left(x_{2}-\frac{5.1}{4\pi^{2}}x_{1}^{2}+\frac{5}{\pi }x_{1}-6\right)}^{2}+10(1-\frac{1}{8\pi })cosx_{1}+10$	[−5,10]^*D*^[0,15]^*D*^
***f*_7_.Eggcrate**	$f_{7}(x)=x_{1}^{2}+x_{2}^{2}+25(sinx_{1}^{2}+sinx_{2}^{2})$	[−10,40]^D^

#### 5.1.2 Experimental results and analysis

Tables 2 and 3 present the performance results of four algorithms with dimensions of 30 and 50, respectively. The evaluation metrics of algorithm accuracy and robustness are measured by the optimal value, worst value, average value, and variance. When the test dimension is 30, it can be observed from the data in the table that the SAOBL-IA algorithm outperforms the other three algorithms in various metrics when applied to functions *f*_*1*_, *f*_*2*_, *f*_*4*_, *f*_*5*_, *f*_*7*_. Among them, for the single-peak function *f*_*1*_ on a sphere, the improvement in performance is particularly evident, and the algorithm’s ability to seek the optimal solution among many local minima is demonstrated by achieving the theoretically best value for function *f*_*4*_. Although the SAOBL-IA algorithm is able to find a relatively optimal solution on function *f*_*3*_, it did not achieve the best results in terms of worst-case value and variance, proving that the improved algorithm has a better ability to escape local optima. And in function *f*_*6*_, SAOBL-IA, IA, and PSO algorithms all achieved the relatively optimal value, but PSO had the best performance according to other standards, which also reflects the potential for improvement in the convergence ability of the SAOBL-IA algorithm.

**Table 2:** Results of 7 benchmarking functions in 30 dimensions.

functions	algorithms	optimum value	worst value	average	standard deviation
*f*_*1*_	PSO	1.1023e-02	3.0274e-01	6.0504e-02	3.2855e-03
	BAS	5.9355e+02	1.1367e+03	8.3709e+02	1.2042e+04
	IA	4.6949e-16	2.6548e+01	1.5893e+00	2.8282e+01
	GWO	1.5722e-18	2.7517e-13	4.0673e-14	7.3695e-27
	SAOBL-IA	**2.4788e-23**	**5.5644e-22**	**9.6004e-23**	**9.4900e-45**
*f*_*2*_	PSO	2.0738e+00	1.4113e+05	2.0243e+04	1.4312e+09
	BAS	1.253e+06	4.4426e+06	2.5773e+06	6.8511e+11
	IA	6.6732e-01	3.1397e+04	2.7273e+03	6.3319e+07
	GWO	8.1583e-01	5.2289e+03	6.5987e+02	5.6489e+06
	SAOBL-IA	**6.6727e-01**	**4.0143e+03**	**4.1898e+02**	**7.4735e+05**
*f*_*3*_	PSO	2.2946e+02	9.3549e+02	3.9000e+02	1.9710e+04
	BAS	6.8539e+02	1.5069e+03	1.0374e+03	3.1126e+04
	IA	1.8262e+02	**2.3364e+02**	2.0100e+02	**1.6112e+02**
	GWO	1.9045e+02	2.4331e+02	2.2253e+02	3.0305e+02
	SAOBL-IA	**1.5099e+02**	3.0204e+02	**1.9987e+02**	5.8031e+02
*f*_*4*_	PSO	1.4622e-03	6.2594e-02	1.6032e-02	2.3371e-04
	BAS	1.1352e+00	1.3115e+00	1.2236e+00	1.5412e-03
	IA	**0**	6.0514e-01	6.9577e-02	2.7737e-02
	GWO	5.5104e-06	1.2279e-03	3.9869e-04	1.0397e-07
	SAOBL-IA	**0**	**0**	**0**	**0**
*f*_*5*_	PSO	1.7324e-01	8.0626e+00	1.7944e+00	3.4248e+00
	BAS	2.7086e+00	3.7420e+00	3.1505e+00	8.2100e-02
	IA	**9.9994e-02**	6.6384e-01	1.9372e-01	1.9860e-02
	GWO	2.6480e-01	6.0779e-01	2.2471e-01	2.0538e-02
	SAOBL-IA	**9.9994e-02**	**5.9306e-01**	**1.3953e-01**	**1.3742e-02**
*f*_*6*_	PSO	**3.9789e-1**	**3.9789e-1**	**3.9789e-1**	**0**
	BAS	**3.9789e-1**	3.544e+2	4.8789e+1	8.146e+3
	IA	3.9937e-1	5.4856e-1	4.2227e-1	7.7916e-4
	GWO	3.9979e-01	1.1821e+00	6.7524e-01	5.8838e-02
	SAOBL-IA	**3.9789e-1**	4.954e-1	4.3037e-1	7.8059e-4
*f*_*7*_	PSO	6.5092e-02	4.7613e+01	1.5011e+01	1.5880e+02
	BAS	5.5007e+02	1.2012e+03	8.7707e+02	2.3988e+04
	IA	7.7656e-17	1.1159e+01	5.0593e-01	4.1237e+00
	GWO	1.5584e—04	4.6077e-+00	1.3685e-01	3.6745e+00
	SAOBL-IA	**3.7854e-23**	**9.4511e-01**	**3.4934e-02**	**2.9785e-02**

**Table 3:** Results of 7 benchmarking functions in 50 dimensions.

functions	algorithms	optimum value	worst value	average	standard deviation
*f*_*1*_	PSO	2.2404e+00	4.9375e+03	8.7302e+02	2.3041e+06
	BAS	9.6375e+02	1.9955e+03	1.4946e+03	5.7349e+04
	IA	7.2518e+00	3.0271e+03	3.2391e+02	3.1918e+05
	GWO	2.3427e-03	1.6852e+02	1.1523e+01	1.1634e+01
	SAOBL-IA	**4.2505e-11**	**1.5009e+01**	**1.3691e+00**	**1.1581e+01**
*f*_*2*_	PSO	1.8837e+05	3.4938e+09	5.3261e+08	7.6544e+17
	BAS	5.3991e+06	1.0804e+07	8.3606e+06	2.3172e+12
	IA	4.8666e+00	2.2223e+07	1.2737e+06	1.8354e+13
	GWO	8.1583e+02	6.6532e+04	7.5893e+03	6.8953e+07
	SAOBL-IA	**9.4123e-01**	**2.2052e+04**	**2.1835e+03**	**2.2157e+07**
*f*_*3*_	PSO	7.9790e+02	1.2359e+04	2.9075e+03	2.9075e+06
	BAS	1.5076e+03	2.2027e+03	1.8876e+03	2.9441e+04
	IA	4.4774e+02	1.7669e+03	7.2504e+02	9.8502e+04
	GWO	3.5986e+02	4.2206e+02	3.9615e+02	5.8399e+02
	SAOBL-IA	**3.5861e+02**	**4.2171e+02**	**3.8703e+02**	**2.4261e+02**
*f*_*4*_	PSO	7.4314e-02	2.7567e+00	1.1866e+00	5.9679e-01
	BAS	1.2376e+00	1.4741e+00	1.3809e+00	2.7456e-03
	IA	3.0685e-01	1.6648e+00	8.7236e-01	8.3516e-02
	GWO	3.9794e-05	1.8510e-03	4.7554e-04	2.6949e-07
	SAOBL-IA	**6.0354e-12**	**3.3298e-04**	**2.7280e-05**	**4.6777e-09**
*f*_*5*_	PSO	8.2482e+00	2.6671e+01	1.7126e+01	1.7706e+01
	BAS	3.5052e+00	4.4501e+00	4.0272e+00	6.9167e-02
	IA	6.2613e-01	5.3779e+00	2.2662e+00	1.2309e+00
	GWO	7.6480e-01	1.1749e+00	9.3581e-01	1.4589e-01
	SAOBL-IA	**1.0124e-01**	**9.4753e-01**	**3.8615e-01**	**2.8978e-02**
*f*_*6*_	PSO	**3.9789e-01**	**3.9789e-01**	**3.9789e-01**	**0**
	BAS	**3.9789e-01**	6.6831e+00	1.1417e+00	3.0340e+04
	IA	3.99889e-01	5.2901e-01	4.2843e-01	1.2696e-03
	GWO	3.9847e-01	6.1795e-01	4.4689e-01	5.2738e-01
	SAOBL-IA	**3.9789e-01**	5.4558e = 01	4.3127e-01	1.5135e-03
*f*_*7*_	PSO	15.722e+01	1.2965e+04	1.8656e+03	9.5419e+06
	BAS	1.1488e+03	1.8362e+03	1.4619e+03	34309e+04
	IA	3.0026e+00	1.3002e+03	2.8885e+02	1.0595e+05
	GWO	2.4753e-03	3.5068e-+03	2.5985e+01	4.6895e+03
	SAOBL-IA	**8.7379e-05**	**5.5909e+01**	**4.1627e+00**	**1.2346e+02**

As the test dimension increases to 50, table data shows that SAOBL-IA algorithm performs better than other algorithms in terms of various indicators in functions *f*_*1*_, *f*_*2*_, *f*_*3*_, *f*_*4*_, *f*_*5*_ and *f*_*7*_. This demonstrates that with the increase in dimension, the improved algorithm has stronger global exploration ability. However, in function *f*_*6*_, both SAOBL-IA and PSO algorithms achieve the best value, but the other criteria are still optimized by the PSO algorithm.

Therefore, based on the experimental data in Tables 2 and 3, although SAOBL-IA algorithm has errors in individual function data, it shows overall better accuracy and robustness compared to the other four algorithms. On the basis of inheriting the advantages of IA algorithm, SAOBL-IA algorithm enhances the global search ability, and reduces the perturbation as the number of iterations increases, which is conducive to local search.

**Fig 5:**
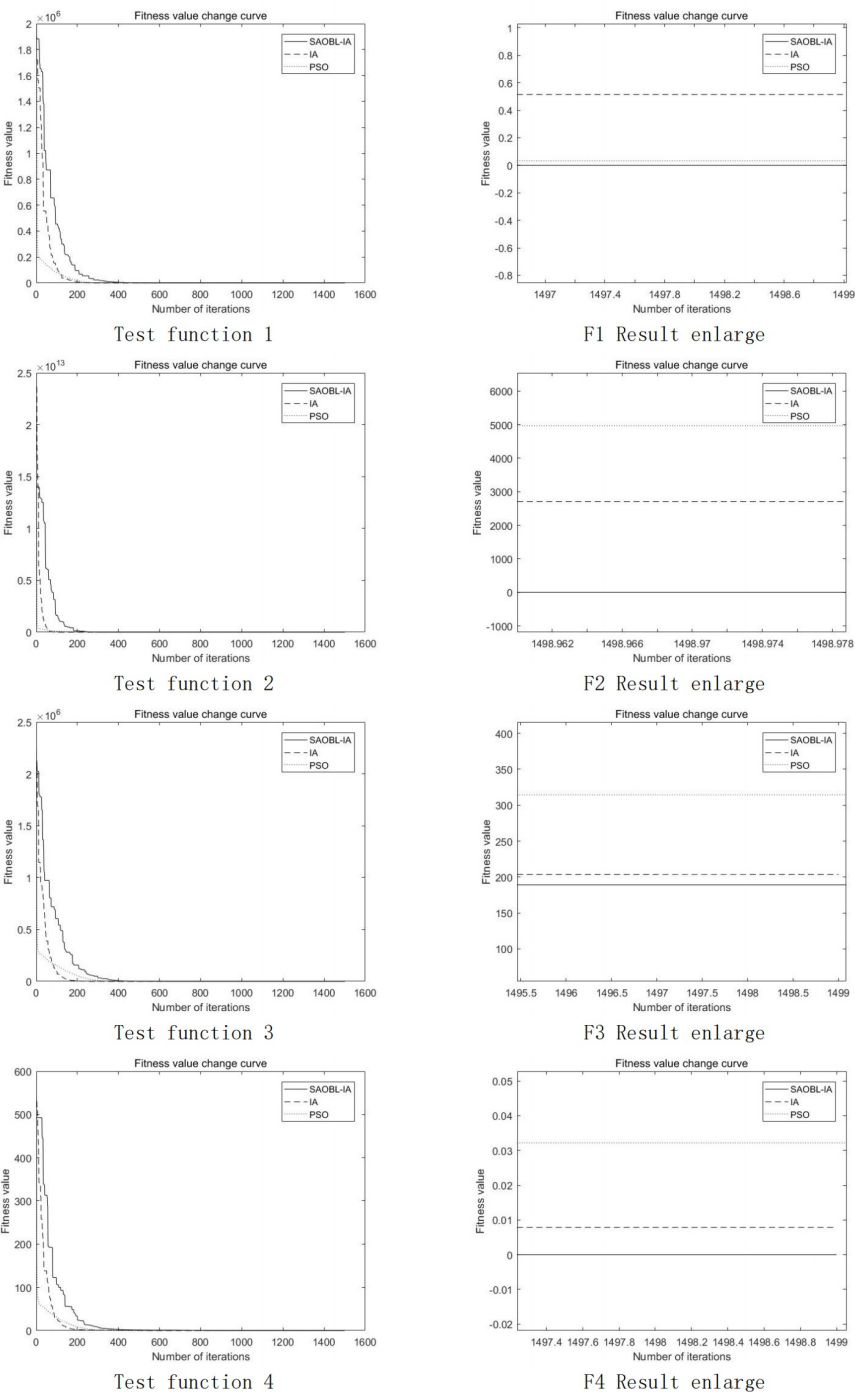
Image segmentation result.

**Fig 6:**
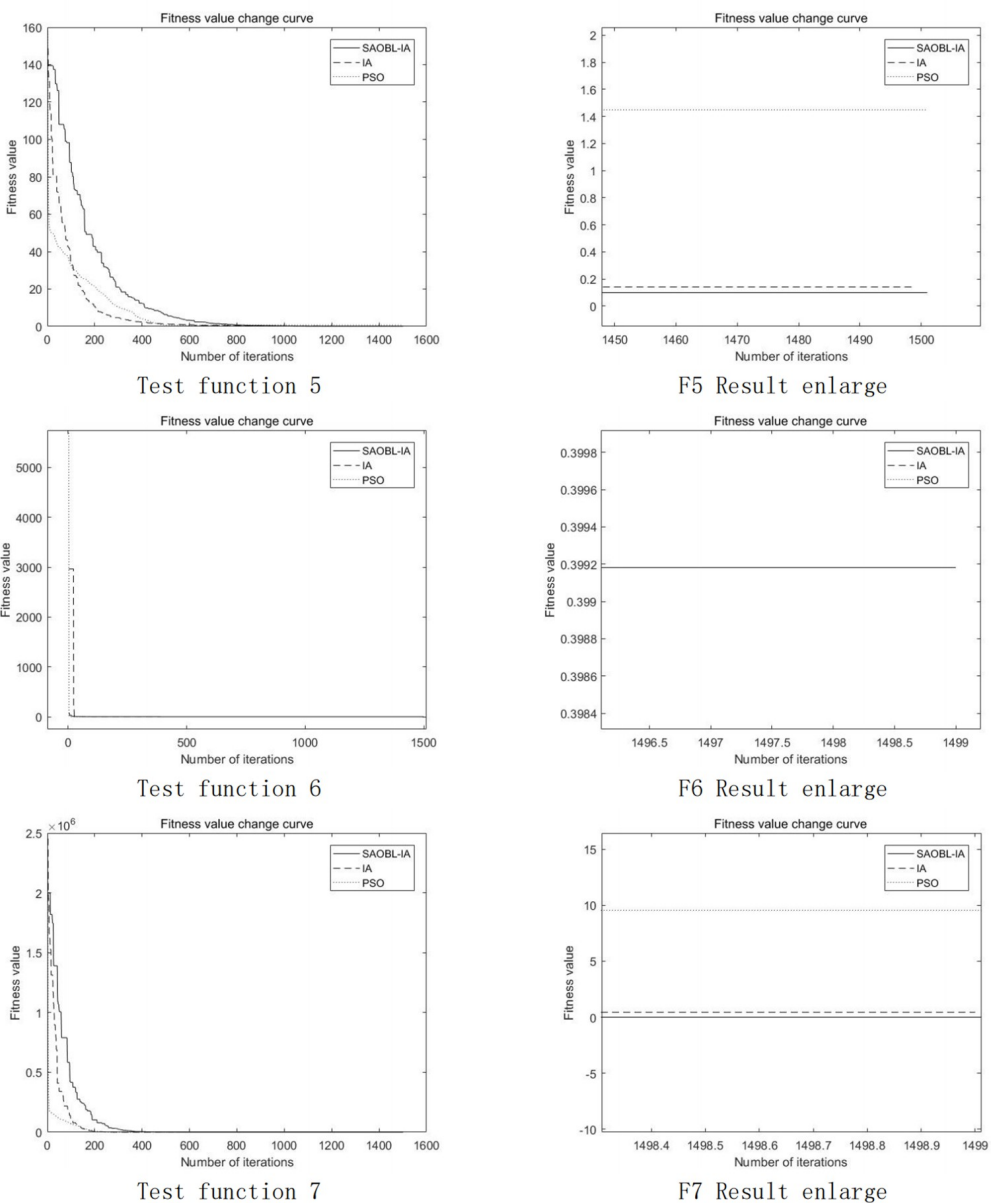
Image segmentation result.

The convergence curves of seven test functions in the comparative algorithm experiments are depicted in Figs 5 and 6 below. Notably, the BAS algorithm is excluded from the plots owing to its distinctive computational approach. Upon analysis of the Fig, it becomes apparent that SAOBL-IA exhibits a slower convergence rate during the early stages compared to the other two algorithms. This phenomenon can be attributed to the simulated annealing algorithm’s inclination to accept suboptimal solutions in the initial phases, thereby facilitating a more thorough exploration of local space. Despite the slower initial convergence, the algorithm accelerates in later stages. Upon scrutiny of the magnified final images, it becomes evident that the enhanced algorithm achieves superior convergence accuracy. Nevertheless, the images also unveil certain limitations of the improved algorithm, particularly in scenarios where obtaining the optimal solution in the initial iterations is imperative, suggesting that the enhancement approach may not be universally applicable.

**Fig 7:**
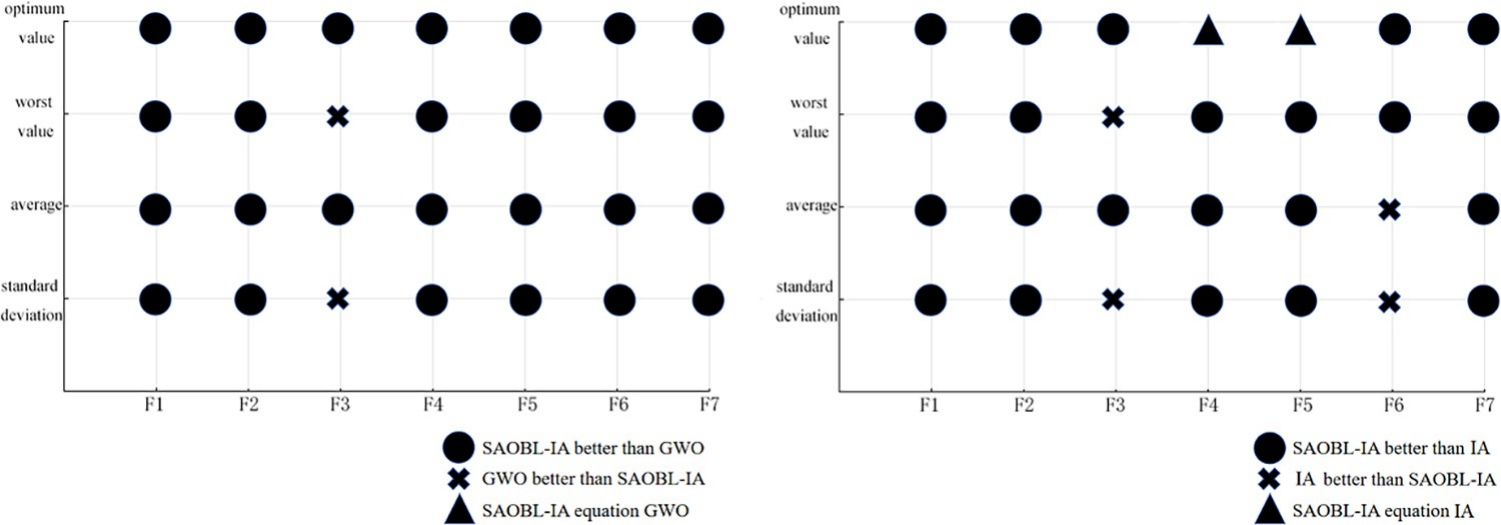
Visualization comparison chart of SAOBL-IA algorithm, and GWO algorithm, IA algorithm (30 dimensions).

**Fig 8:**
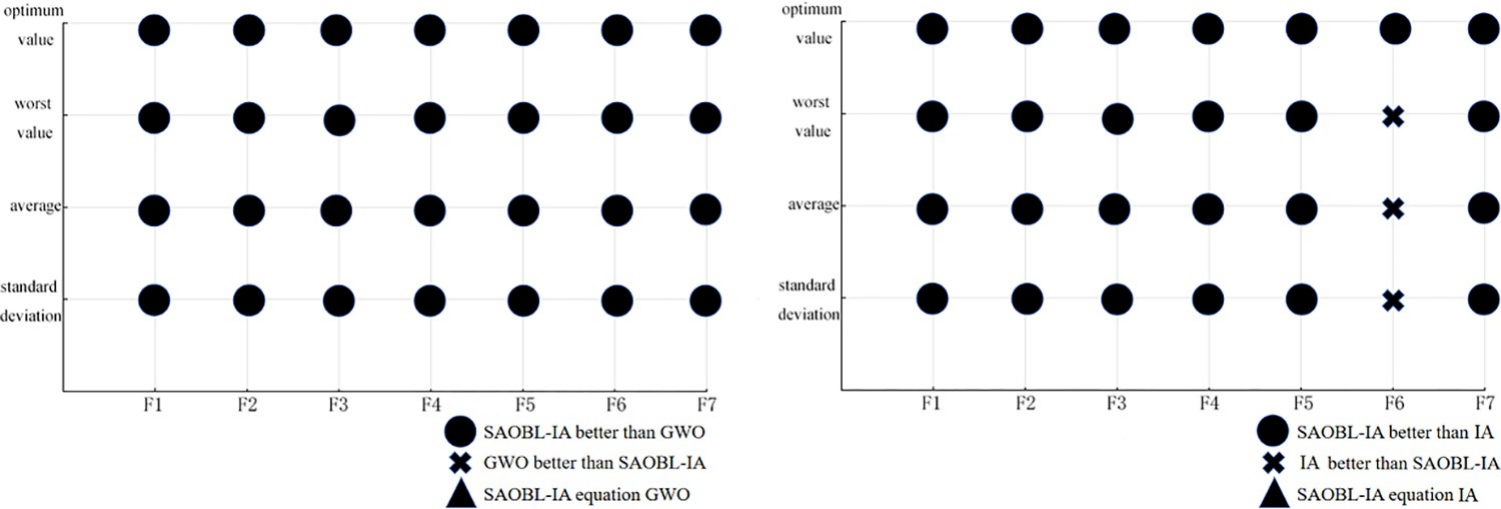
Visualization comparison chart of SAOBL-IA algorithm, and GWO algorithm, IA algorithm (50 dimensions).

We visualized a part of the statistical data in Tables 2 and 3 (see Figs 7 and 8). For a detailed analysis of these statistical data, please refer to the "Experimental results and analysis" section. Figs 7 and 8 visually demonstrate the comparison results of the SAOBL-IA algorithm proposed in this paper and GWO algorithm, IA algorithm on 7 test functions. This provides a clearer explanation of the convergence and robustness of the SAOBL-IA algorithm on various test functions. These visual comparisons can provide a intuitive understanding of their performance under different conditions.

### 5.2 Experimentation and analysis of lung image segmentation algorithm

To verify the performance of the Adaptive bifurcation 2D OTSU Lung Image Segmentation based on SAOBL-IA algorithm. The open source COVID-CT Dataset was selected as the dataset to test on CT images of COVID-19 pneumonia, the algorithms are all in Matlab R2016a software for Windows 10 with an i7-11800H 2.3GHz CPU and 16GB of RAM.

#### 5.2.1 Experimental results and analysis

To assess the performance of the proposed method, we conducted a comparative analysis with the traditional 2D OTSU method and the approach described in reference [33] when evaluating the image segmentation capability. Furthermore, for the assessment of segmentation speed, we integrated an algorithm combining Particle Swarm Optimization (PSO) with the traditional 2D OTSU method, both of which are intelligent algorithms. To ensure experimental fairness, we maintained uniformity in the basic parameters across all four algorithms.

**Fig 9:**
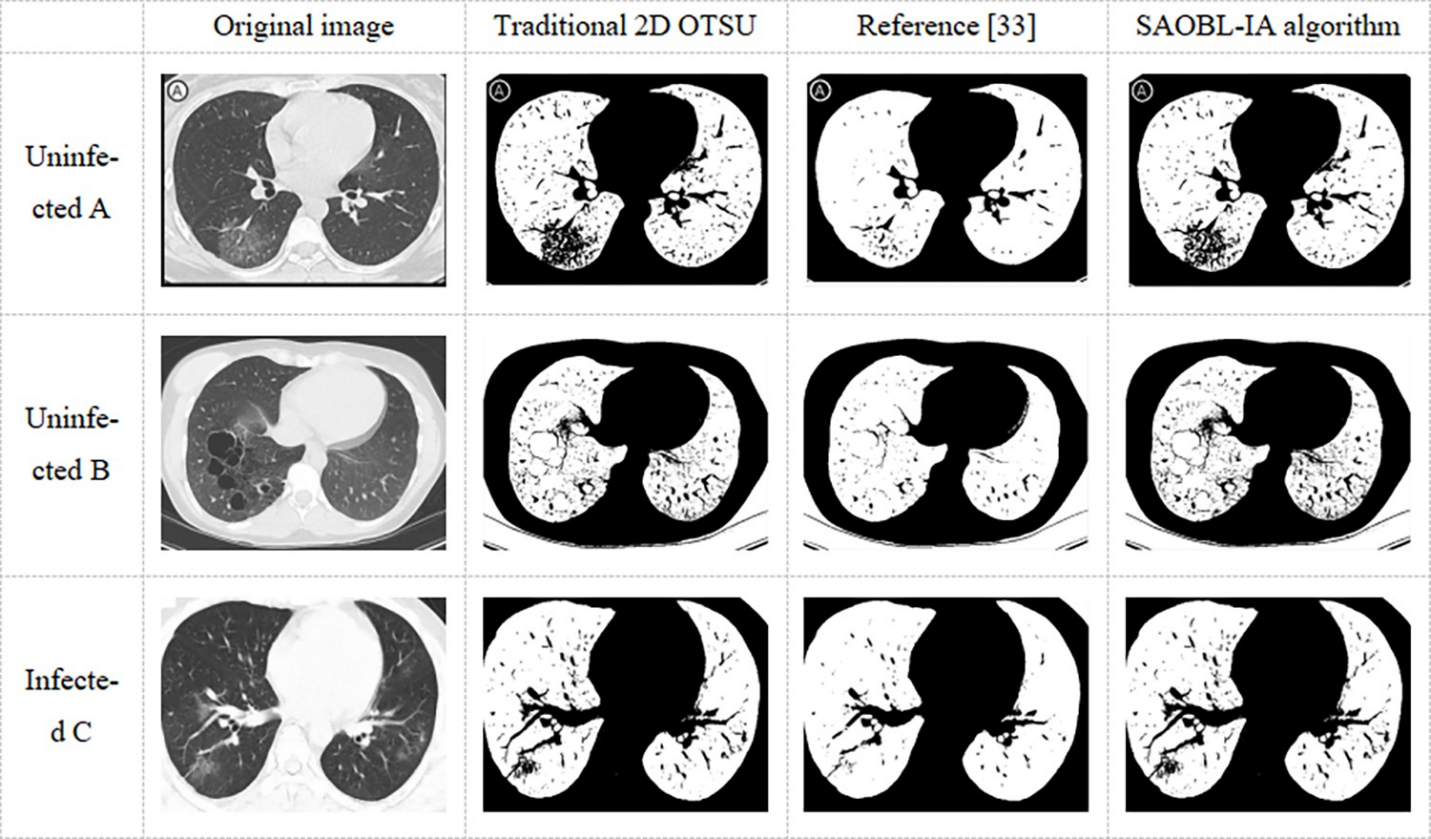
Image segmentation result.

The experimental results of the segmentation are shown in Fig 9 from left to right: original image, traditional 2D OTSU, reference [33], and the proposed algorithm. From top to bottom: uninfected individual A, uninfected individual B, and infected individual C with COVID-19.

From Fig 9, it can be observed that although the traditional 2D OTSU method provides finer segmentation, it still fails to handle certain types of noise adequately. On the other hand, the approach described in reference [33] takes into account background and noise considerations more comprehensively, but its segmentation is still significantly affected. In contrast, our proposed algorithm demonstrates finer details while disregarding noise interference. However, the segmentation results for the infected individual C with COVID-19 are consistent with the traditional 2D OTSU method. This can be attributed to the fact that the neighborhood grayscale mean values in that region are too close to the boundary, making it difficult to achieve a clear bifurcated. To provide a more intuitive representation of the performance differences, this study employed the Peak Signal Noise Ratio (PSNR) [38] as an objective measure of image quality evaluation. PSNR is defined based on the Mean Square Error (MSE) as follows:

$$PSNR=10\;\log_{10}\left(\frac{{MAX}_{I}^{2}}{MSE}\right)$$ (23)

$$MSE=\frac{1}{mn}\sum_{i=0}^{m-1}\sum_{j=0}^{n-1}{\left[I(i,j)-K(i,j)\right]}^{2}$$ (24)

Here, the original image I has a size of m*n, K represents the segmented image, MAX_I_ is the maximum pixel value of the image, and PSNR is measured in dB. If each pixel is represented by 8 bits in binary, then the value is calculated as follows: 2^8^−1 = 255.

The data in Table 4 indicates that our algorithm exhibits superior performance in terms of PSNR compared to the traditional 2D OTSU method and the approach outlined in reference [33], thereby revealing more detailed information from the original images. Additionally, Table 5 presents a comparison of the runtime among the algorithms, clearly demonstrating a significant advantage of our proposed algorithm.

**Table 4:** Segmented image PSNR value.

Image	Traditional 2D OTSU PSNR	Reference [33] PSNR	SAOBL-IAPSNR
Uninfected A	3.8017	3.7058	3.8419
Uninfected B	3.8236	3.7508	3.8641
Infected C	3.0724	3.0258	3.0724

**Table 5:** Segmented image running time.

Image	Traditional 2D OTSU	Reference [33]	Pso 2D OTSU	SAOBL-IA
	running time(s)	running time(s)	running time(s)	running time(s)
A	108.3113	104.5270	8.5725	7.9164
B	100.6139	123.8258	9.5942	8.3410
C	105.7958	109.8922	8.9715	7.4519

## 6. Conclusions and future works

The main work of this paper includes the following three parts:

1. Presents an improved Island Algorithm based on the Simulated Annealing and Opposition-based Learning (SAOBL-IA). By incorporating opposition-based learning during the balancing phase to enhance the algorithm’s search breadth and speed; introducing simulated annealing during iteration to prevent convergence to local optima while further narrowing the search space and increasing the convergence rate. The improved algorithm has addressed the issues of incomplete local search and susceptibility to local optimal solutions compared to the original algorithm. Experimental results also demonstrate that the improved algorithm exhibits better search capabilities for global optimal solutions and enhanced robustnes.
2. To address the issue of misclassified pixels in noise and background regions on the conventional 2D OTSU histogram, proposes a modified Adaptive Bifurcation 2D OTSU approach, the AB2D-OTSU incorporates two line segments at the threshold points and adaptively adjusts the slope of the lines based on the proportion of misclassified pixels, thereby achieving a reasonable classification of the background and target regions.
3. In response to the slow segmentation speed of the original algorithm, proposes an image segmentation method combining the SAOBL-IA and the AB2D-OTSU, and apply it to lung image segmentation. The comparative analysis of experimental results shows that the new image segmentation algorithm avoids the tedious search process of traditional methods, significantly accelerates the speed of image segmentation, and achieves higher segmentation accuracy.

Overall, the island algorithm based on simulated annealing and opposition-based learning has made significant progress in experimental results. However, the introduction of two improvement methods has enhanced the complexity of the algorithm compared to the original island algorithm, resulting in issues such as increased computation time. Additionally, due to the algorithm’s characteristics, it may be difficult to find the global optimal solution when faced with test functions and industrial optimization problems with a valley shape characterized by high sides and a low middle. To address these issues, future research will consider using optimization methods with lower complexity to improve the island algorithm, aiming to reduce algorithm complexity and enhance overall efficiency. Furthermore, individual studies will be conducted for specific test functions. Meanwhile the deep integration of technologies such as big data and artificial intelligence with healthcare will greatly improve the efficiency of diagnosis, surgery, and public health management [39]. Therefore, in later stages, we also consider introducing the Q-learning method from reinforcement learning, treating each plant as an independent agent, and conducting personalized evolution based on its position and reward mechanism to better harness the algorithm’s usability and optimization accuracy.
